# Optimum Design and Application Research of Eddy Current Sensor for Measurement of TBM Disc Cutter Wear

**DOI:** 10.3390/s19194230

**Published:** 2019-09-29

**Authors:** Fei Wang, Changhao Men, Xiangwei Kong, Lingxiang Meng

**Affiliations:** 1 School of Mechanical Engineering and Automation, Northeastern University, Shenyang 110819, China; 2 Key Laboratory of Vibration and Control of Aero-Propulsion System Ministry of Education, Northeastern University, Shenyang 110819, China; 3 Institute of Engineering Technology, University of Science and Technology Beijing, Beijing 100083, China; mch_41040193@163.com; 4 China Construction Eighth Engineering Division Rail Transit Construction Co.,Ltd. Nanjing 210046, China; menglxzjbj@163.com

**Keywords:** disc cutter wear, eddy current sensor, Ansoft Maxwell simulation, optimization design

## Abstract

In view of the fact that the mature eddy current sensors on the market have a small measuring range and a certain requirement for the measured conductor area, which cannot meet the requirement of TBM disc cutter Wear monitoring, Ansoft Maxwell simulation was used to optimize the coil geometry parameters and circuit structure of eddy current sensor in this study. A special sensor for measuring disc cutter wear was designed, and the feasibility of the design was verified by measuring a 17-inch disc cutter. The results indicate that the linear range of the eddy current sensor is 35 mm under the condition that the measured blade width is 19 mm, which can meet the requirement of disc cutter wear measurement and provide monitoring data for real-time disc cutter wear monitoring system.

## 1. Introduction

A Tunnel Boring Machine (TBM) is a set of mechanical equipment which integrates the functions of excavation, slag removal, lining and control [1]. It is called the “aircraft carrier” of construction machinery. The disc cutter is used as a rock-breaking tool in the TBM excavation work. If the single cutter reaches the wear limit and the tool is not replaced in time, further drilling operation will lead to an increase in the adjacent tool wear. Cascade failure of all cutting tools is the worst case. It not only damages tool components, but also takes a lot of time to change the tool. Serious cascade failure may cause cutter-head damage [2].

At present, the main method of TBM cutter wear detection is manual inspection after shutdown. The cabin door is opened, and the staff enters the front of the cutter-head to check and replace the cutter after the TBM is shut down for more than 24 h. Although this method is simple and reliable, it seriously affects the construction efficiency and requires a high labor cost. Moreover, opening the door is accompanied by very high risk, which may cause the collapse of the excavation surface and even casualties. According to the practice statistics of a tunnel project, the cost of cutter accounts for 1/5 to 1/3 of the project cost, and the time of changing cutter accounts for about 1/3 of the construction time. The number of replaced cutter rings reaches 80% due to wear and tear. Therefore, to improve tunneling efficiency and reduce cost, real-time monitoring of cutter wear is a technical problem to be solved urgently.

Because of different geology, the wear mode and wear rule of TBM cutter are different in the working process. In the process of TBM advancing forward, the cutter-head and cutter themselves are in a continuous rotating state, and some influencing factors, such as cutting rock, soil and water in the working environment, greatly interfere with the study of the wear degree of TBM tools. Most of the research currently focuses on the prediction of cutter wear, while some scholars and tunneling equipment manufacturers are conducting on-line wear detection research. The main methods include adding odorant, tunneling parameter analysis, rock slag shape analysis, manual inspection after shutdown, tool wear induction device and so on.

To study the tool wear of rock in TBM tunneling process, the abrasiveness and strength of rock are generally used as the basis for predicting the wear life of cutter abroad, such as ABR index test of French LCPC model and CAI index test of CSM model of Colorado Institute of Mining and Metallurgy in the United States [3,4]. During the construction of Qinling Tunnel, the cutter with MOLYUAN was used by WIRTH [5]. American Robbins Corporation developed a monitoring system based on wireless transmission by collecting three state indicators of cutter speed, temperature and vibration [6].

The wear of TBM cutter has also been studied by Chinese scholars. Zhu [7] performed research on acoustic emission signal of TBM cutter based on improved CRITIC method to evaluate and predict tool wear status. Zhang [8] designed an on-line monitoring device to judge cutter wear by using the method of laser optical path on-off. Liu [9] reformed the cutter base and calculated the wear amount of the cutter according to the corresponding relationship between voltage and wear. Tan [10] designed a rotational speed monitoring device for TBM cutters in his patent. The relationship between the measured rotational speed of disc cutters detected by sensors and the preset reference value (theoretical rotational speed of cutters) is used to judge the running state and wear state of disc cutters.

In summary, regarding the real-time monitoring of cutter wear, some manufacturers are in a state of technical blockade, and the papers published by university and research institutes do not specifically introduce what kind of sensors to use, how to arrange sensors, and how to transmit data, just a general overview. Most of the relevant research are in the process of scientific study and exploration, mainly focusing on the patented structure and prediction of wear measurement, but no cutter wear monitoring device has been found to be applied in actual tunnel engineering.

In the process of TBM excavation, the cutter extrudes and cuts rocks, with violent vibration, slag splashing, and even with impurities such as mud and water. The working environment is poor. Therefore, non-contact sensor with good anti-interference should be selected for cutter wear monitoring. Thus far, the commonly used non-contact sensors in engineering [11,12] mainly include laser sensor, capacitive sensor, eddy current sensor and so on. The characteristics of three commonly used non-contact displacement sensors are compared as shown in Table 1.

Eddy current sensor system is widely used in displacement and vibration measurement in practical engineering because of its unique advantages. Li [13] measured the axial displacement of maglev rotor by eddy current sensor. Ding [14] detected surface displacement by eddy current sensor. Xia [15] adopted eddy current sensor to detect the speed and mileage of railway locomotive. Wu [16] used eddy current sensor to measure the relative distance between the electromagnet and track of maglev train.

By comparing the characteristics of three commonly used non-contact displacement sensors, combined with the working environmental characteristics of the cutter during TBM excavation process, eddy current sensor is chosen to measure the cutter wear. Due to the effect of eddy current on the surface of cutter ring, the change in the output voltage value ultimately reflects the change in the relative distance between the probe end face and the cutter ring.

At present, the most commonly used cutters in tunnel engineering are 17-inch in diameter. Eighteen-inch and 20-inch cutters have been produced. The measured area of the 17-inch cutter ring is 19 mm, and the wear limit is 20 mm. The tool can be changed when the wear amount reaches 15–18 mm. Considering the installation distance between eddy current sensor and cutter ring, the measurement range of eddy current sensor needs to reach 30–40 mm. However, the range of mature eddy current sensors on the market is 10–20 mm, and the sensor has a good linearity when the measured conductor area reaches 2–3 times the probe diameter. Therefore, this study optimized the coil geometry parameters such as inner diameter, outer diameter, thickness, turns and circuit of eddy current sensor by Ansoft Maxwell simulation. In the case of insufficient measured area, a large range and good linearity eddy current sensor was designed to detect the wear of cutter ring. The developed eddy current sensor was used to measure the 17-inch cutter to verify the feasibility of the design and various performance indicators.

## 2. Working Principle of Eddy Current Sensor

**Figure 1:**
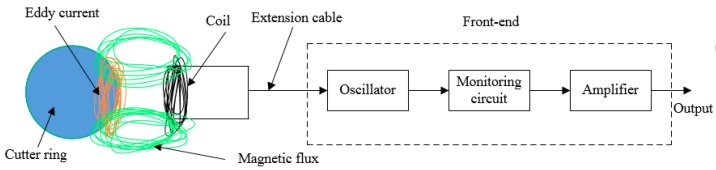
Eddy current sensor structure.

The structure of the eddy current sensor is composed of a detection coil, an extension cable and a front-end, as shown in Figure 1. The detection circuit consisting of oscillating circuit, detector and amplifier circuit is completely enclosed in the metal box for the purpose of shielding interference signals from the outside world. 

**Figure 2:**
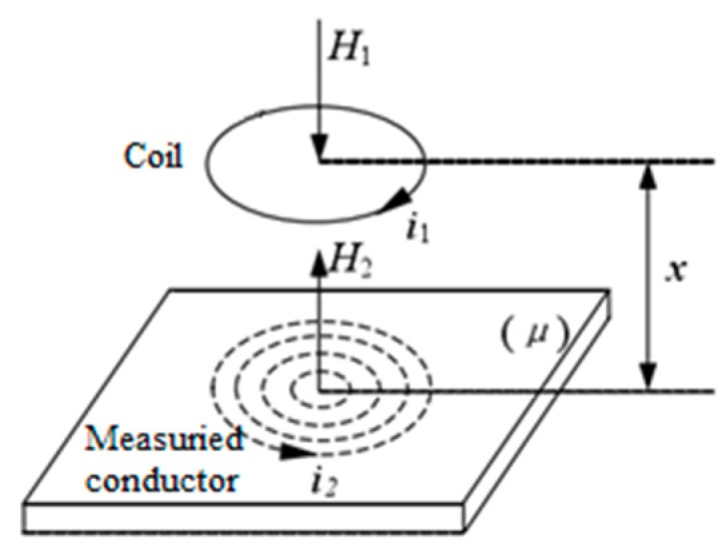
The eddy current reaction.

When the eddy current sensor passes current $i_{1}$, a sinusoidal alternating magnetic field $H_{1}$ is generated around the coil. A closed spiral induced current $i_{2}$ is generated on the surface of the cutter ring in the magnetic field, which is called the eddy current. The eddy current $i_{2}$ on the surface of the cutter ring simultaneously generates a new alternating magnetic field $H_{2}$, which is opposite to the direction $H_{1}$. Since the reaction of the magnetic field $H_{2}$ reduces $H_{1}$, the impedance of the probe coil changes accordingly. The reaction of the eddy current is shown in Figure 2. The change of the equivalent impedance of coil is directly related to the excitation frequency of the coil, the relative position between the probe coil and the cutter ring, the conductivity of the cutter ring, and the magnetic permeability [17].

## 3. Three-Dimensional Finite Element Simulation

The real-time monitoring of TBM cutter wear is essentially measuring the change in the distance between sensor probe and the cutter ring. The sensor measures the distance, ideally with greater linearity and higher sensitivity. The linearity and sensitivity of eddy current sensor are mainly affected by the distribution of coil magnetic field, which is also affected by the material and the area of measured conductor, excitation signal, coil parameters and other factors [18]. At present, there is no research on TBM cutter as the measured conductor. To improve the accuracy of wear measurement, it is necessary to explore various factors to provide a basis for the design of TBM cutter wear monitoring sensor.

This simulation was based on Ansoft Maxwell, a special simulation software for electromagnetics. The range of simulation measurement was 0–40 mm, that is, the relative distance between the probe of eddy current sensor and the cutter ring was 0–40 mm. To improve the accuracy of measurement, a simulation was made from 0 to 2 mm, and under different coil parameters.

### 3.1. Setting of Solution Domain

The essence of finite element simulation analysis and calculation is to solve the matrix equation, which has innumerable sets of solutions. However, the solution that satisfies both the equation and its boundary conditions is unique. The boundary condition is the fixed value set on the boundary of the simulation model. This condition is the premise that the matrix equation has a unique solution.

Ansoft Maxwell software is divided into six different solvers according to common computing models. The magnetic field includes static magnetic field, eddy current field and transient magnetic field. The electric field includes electrostatic field, AC conduction field and DC conduction field. In this study, the eddy current sensor was used to measure cutter wear. The relationship between the impedance or inductance and the distance between the sensor and the cutter ring was obtained by simulation. Therefore, the type of domain solver was chosen as eddy current field.

### 3.2. Creation of Geometric Model

**Figure 3:**
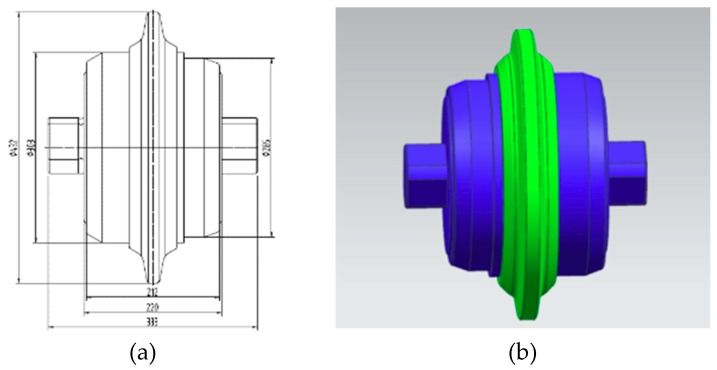
Seventeen-inch cutter: (**a**) two-dimensional drawing; and (**b**) three-dimensional model.

In this study, the wear of 17-inch cutter was measured, thus the prototype of 17-inch cutter was simulated as the measured conductor. A three-dimensional model was drawn according to the two-dimensional drawing 1:1 of the 17-inch cutter, as shown in Figure 3. The cutter wear is actually the wear of the cutter ring, thus it was only necessary to import the part of the tool ring into Ansoft Maxwell software. A 17-inch three-dimensional cutter model was established in UG 9.0 and imported to Maxwell software in sat format. The sensor model needed to be adjusted at any time according to the simulation results, thus it was drawn in Maxwell software.

### 3.3. Setting of Solution Domain and Material Parameters

**Figure 4:**
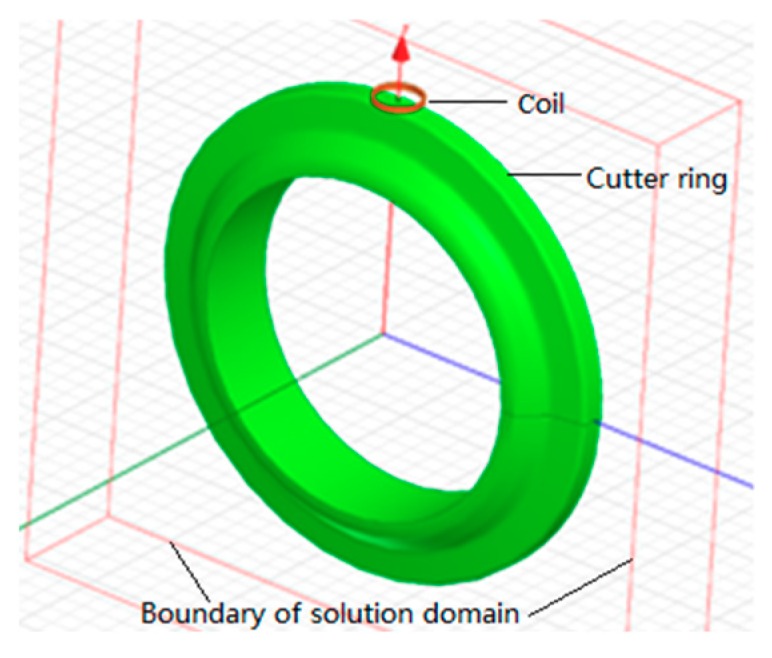
Simulation model.

When solving eddy current field in Maxwell software, the range of the solution domain can include the model of cutter ring and sensor. On the basis of the model, the space of *x*, *y*, and *z* axes plus 20 mm can be used as the solution domain, as shown in Figure 4.

The cutter ring, sensor coil and solution domain were included in the simulation. In this simulation and the following experiments, the domestic 17-inch constant cross-section cutter was used. The material of the cutter ring is 40CrNiMo. The sensor coil is made of copper wire. The solution domain is vacuum. The material parameters [19] are shown in Table 2.

### 3.4. The influence of Coil Parameters on the Sensor

Coil geometric parameters include inner diameter, outer diameter, thickness, turns and frequency. Low frequency transmission eddy current sensors are generally used for thickness measurement. High frequency reflective eddy current sensors are commonly used to measure displacement or vibration. Too high frequency not only reduces the linearity, but also increases the difficulty and time of simulation. Therefore, combined with the existing sensors, the coil excitation frequency in this study was selected to be 1 MHz. Based on many simulations, the following typical parameters were selected for explanation.

**Figure 5:**
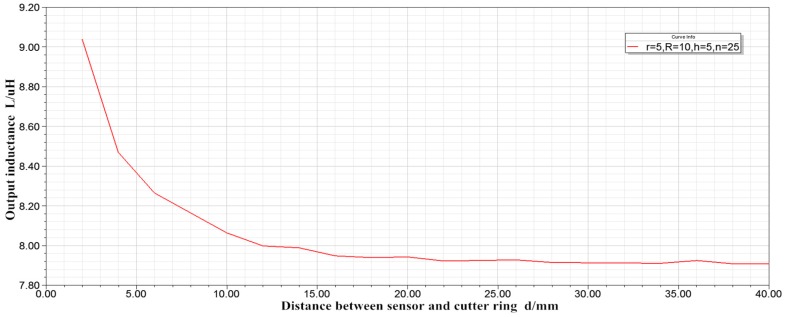
Characteristic curve of inductance displacement.

**Figure 6:**
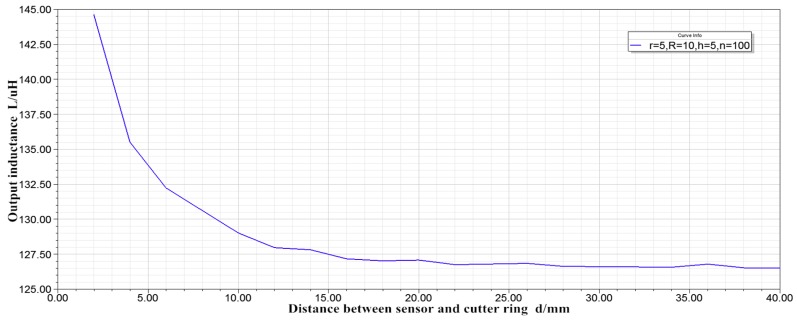
Characteristic curve of inductance displacement.

(1) With internal radius r = 5 mm, external radius R = 10 mm, thickness h = 5 mm, and turn number n = 25, the inductance displacement characteristic curve is shown in Figure 5

(2) With internal radius r = 5 mm, external radius R = 10 mm, thickness h = 5 mm, and turn number n = 100, the inductance displacement characteristic curve is shown in Figure 6.

Comparing Figure 5 and Figure 6, it can be obtained that, when n = 25 and L = 7.91–9.04 μH, and, when n = 100 and L = 126.54–144.63 Μh, the linearity of the two curves is basically the same. In other words, when the inner radius, outer radius and thickness of the coil are constant, increasing the number of winding turns can only increase the sensitivity of the sensor, and has little effect on the linearity of the sensor. When the distance between the sensor and the cutter ring exceeds 22 mm, the output inductance remains basically unchanged. Therefore, a sensor with an inner radius r = 5 mm, an outer radius R = 10 mm, and a thickness h = 5 mm cannot satisfy the cutter wear measurement.

**Figure 7:**
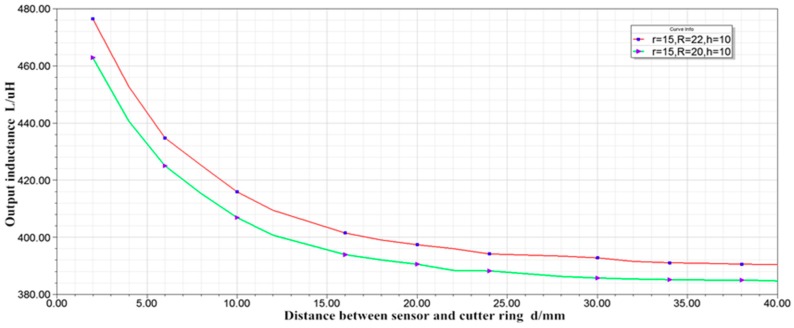
Characteristic curve of inductance displacement.

(3) With inner radius r = 15 mm, outer radius R = 20 mm, and thickness h = 10 mm and with inner radius r = 15 mm, outer radius R = 22 mm and thickness h = 10 mm, the simulation results are shown in Figure 7.

By comparing the two curves, it can be concluded that, when the inner radius, thickness and turns of the coil are constant, the inductance increases with the increase of the outer radius. In other words, increasing the outer radius can improve the sensitivity of the sensor.

The curve in Figure 7 is much smoother than that in Figure 6. That is, increasing the inner radius can improve the linearity of the sensor.

**Figure 8:**
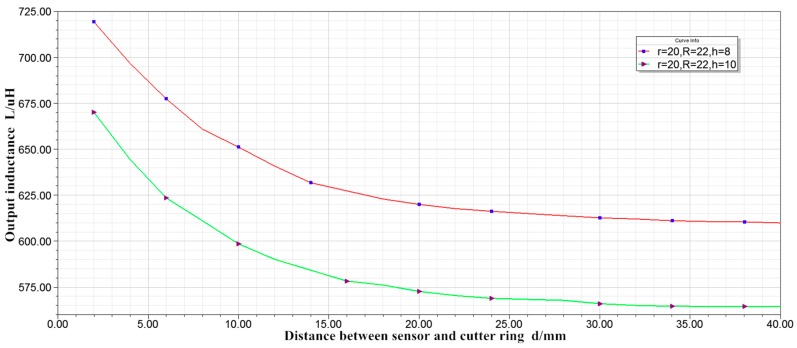
Characteristic curve of inductance displacement.

(4) With inner radius r = 20 mm, outer radius R = 22 mm, and thickness h = 8 mm and with inner radius r = 20 mm, outer radius R = 22 mm and thickness h = 10 mm, the simulation results are shown in Figure 8.

By comparing the two curves, it can be concluded that, when the inner radius, outer radius and number of turns of the coil are constant, the inductance increases with the decrease of the thickness. That is, the smaller is the thickness, the greater is the sensitivity of the sensor. The trend of the two curves is basically the same, thus the change of thickness has little effect on the linearity of the sensor.

The curve in Figure 8 is much smoother than that in Figure 7, that is increasing the inner radius can improve the linearity of the sensor.

To sum up, when the inner radius of the coil is constant, the sensitivity of the eddy current sensor can be improved by increasing the number of turn or the outer radius and decreasing the thickness. Increasing the inner radius of the coil can improve the linearity of the eddy current sensor. To make the sensor reach 40 mm and have high linearity, the sensor for measuring cutter wear needs to be designed as a thin coil with large inner diameter.

## 4. Circuit Design of Eddy Current Sensor

Eddy current sensor output impedance Z, inductance L, and quality factor Q were investigated. Output voltage or current value is required in actual measurement. Therefore, it is necessary to match the corresponding circuits. Matching measuring circuits can be divided into three types: constant frequency amplitude modulation sensor, variable frequency amplitude modulation sensor and frequency modulation sensor.

**Figure 9:**
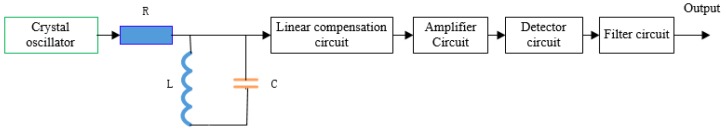
Schematic diagram of circuit structure.

In terms of linearity range, frequency conversion amplitude modulation is better than constant frequency amplitude modulation. In terms of sensitivity, frequency conversion amplitude modulation and constant frequency amplitude modulation are better than frequency modulation. In terms of stability, the constant frequency amplitude modulation of quartz crystal oscillators has the best performance, which can eliminate the shortcoming of small change of output variable caused by frequency instability [20]. In the process of TBM work, the environment is complex and harsh, and there are many interference factors. Therefore, a constant frequency amplitude-modulated eddy current sensor was designed, and its circuit structure is shown in Figure 9.

**Figure 10:**
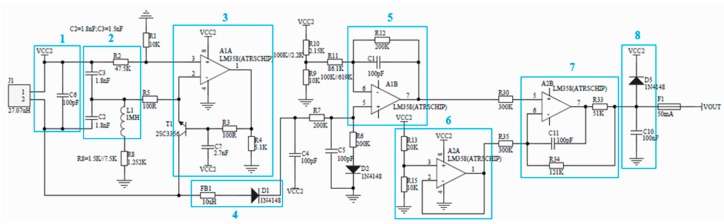
Parameters of the design sensor.

The series resonant circuit is usually used in the transmitting circuit. Its advantage is that the circuit is simple and the influence of coil internal resistance is not considered. The disadvantage is that the output voltage changes are also small, which is not conducive to subsequent processing. The parallel resonant circuit can obtain a large output voltage change value by appropriately adjusting the resistance of the voltage dividing resistor, which is beneficial for subsequent processing [21,22]. Therefore, LC parallel oscillator was chosen in this study. Based on the Multisim simulation, reference to the existing circuit knowledge, the specific circuit structure, model and parameters of the design sensor are shown in Figure 10.

In Figure 10, Circuit 2 is the signal source and the signal processing sequence is from left to right. J1 is the coil of the eddy current sensor. Box 1 shows a linear trimming circuit that compensates for the nonlinearity of the eddy current sensor. Box 2 shows an LC oscillating circuit formed by C2, C3 and L1, which generates a 1-MHz sine wave excitation signal input to an effective component such as an eddy current sensor probe, and is a signal source of the entire circuit. Box 3 shows the bias circuit and signal conditioning circuit, which acts as the feedback loop of Circuit 2 and maintains the continuous oscillation output of LC. Box 4 shows a rectifier circuit, which converts the eddy current sensor signal generated by LC oscillation into a single voltage signal, and outputs it to the input end of the post-stage op-amp circuit for signal comparison and amplification. Box 5 shows a differential signal amplifier circuit, which amplifies and filters the rectified voltage signal and outputs it to the later stage. Box 6 shows a reference voltage follower, which compares with the output voltage produced by Circuit 5 and outputs the required sampling signal. Box 7 shows a signal conditioning circuit that adjusts the blind area and range of the sensor, changes the output voltage, and transmits useful sampling signals to the output port. Box 8 shows output filter and protection circuit. The capacitor filters the output signal to reduce high frequency interference. The diode is a voltage clamping device to prevent the output voltage from exceeding the limit and protect the post-stage equipment or devices.

**Figure 11:**
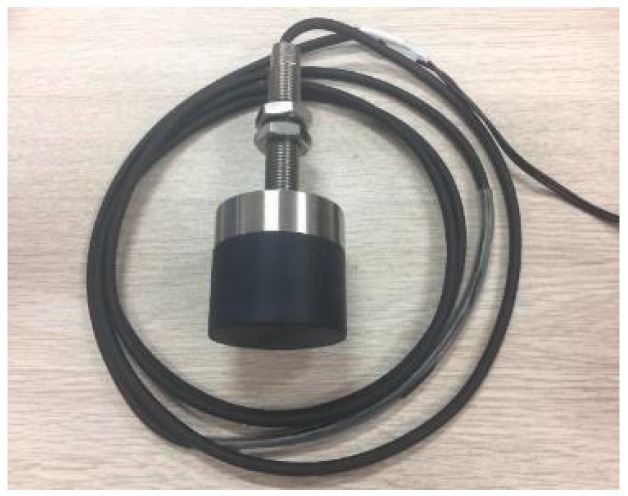
The developed eddy current sensor.

Based on Ansoft Maxwell simulation, combined with LC oscillator (excitation frequency 1 MHz and matching capacitor 1.8 nF), detector (1N4148), filter (output capacitor 100 nF) and operational amplifier (LM358), the external form of developed eddy current sensor is shown in Figure 11. 

The specific parameters of the developed eddy current sensor are shown in Table 3.

## 5. Case Study

Relevant research shows that eddy current sensor has requirements for the measured material and area. The voltage values measured by different materials are different, and the voltage values measured by different areas are different. To find the relationship between voltage and wear accurately, a 17-inch cutter was used in this experiment. Due to the temporary shortage of 17-inch cutter in the laboratory, a 17-inch constant cross-section cutter was measured for this experiment in a rail transit company. The material of the cutter ring is 40CrNiMo, and the width of the cutter edge is 19 mm, which is exactly the same as the cutter parameters used in the simulation presented above.

**Figure 12:**
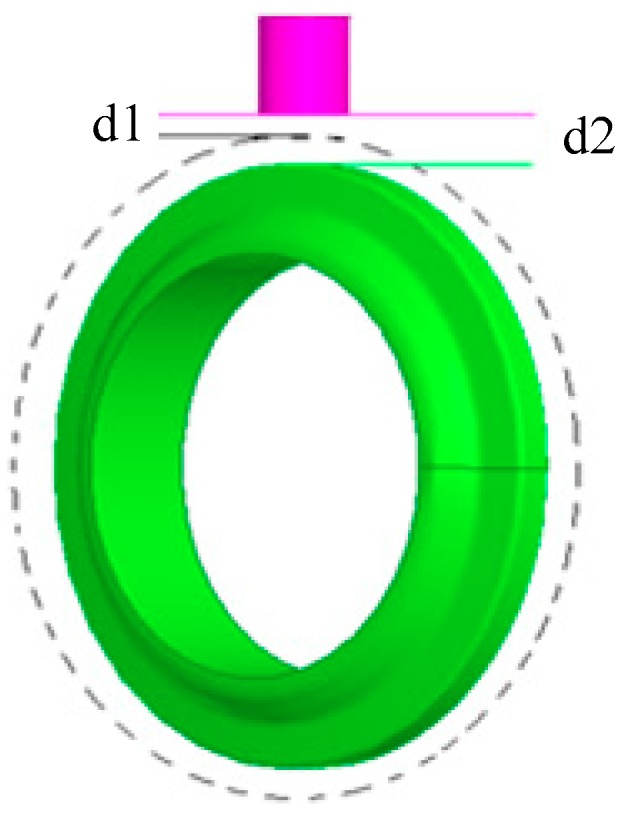
Principle of wear measurement.

During the TBM tunneling process, the cutter squeezes rock and the wear is a slow process. With the cutter ring wears, the diameter of the cutter gradually decreases, and the distance between cutter ring and eddy current sensor also increases. The wear volume is the change of the distance between the cutter ring and the probe of eddy current sensor, as shown in Figure 12. The wear amount d = d2−d1. d2 is the distance between probe of the eddy current sensor and the worn cutter ring. d1 is the distance between probe of the eddy current sensor and the cutter ring before wear. It is the installation distance, thus d1 is a constant.

Due to the limitation of experimental conditions, the 17-inch cutter was fixed in this experiment. The eddy current sensor probe faced the cutter ring, and the distance was from 0 to 49 mm. The measurement was performed with a variation of 1 mm. The experimental data are shown in Table 4.

When the distance between the probe of eddy current sensor and the cutter ring was 43 mm, the output voltage reached the maximum value and did not change, i.e. the range of sensor was 43 mm. When the distance between the sensor and the ring was more than 35 mm, the change of output voltage was very small, i.e., the linear range of the sensor was 35 mm. 

Regarding the choice of the range of data fitting, firstly, when the distance exceeded 35 mm, the sensor was close to the range of measurement, the data were not sensitive and the measurement was not accurate. Secondly, considering the data after 35 mm, the complexity of curve fitting increased and the program ran slowly. Finally, the initial distance between the eddy current sensor and the cutter ring was set at 5 mm, and the ultimate wear of the 17-inch cutter was 20 mm, leaving a space of 10 mm. Therefore, the data fitting range was selected to be 0–35 mm.

Let the fitting model function be polynomial as

(1) $$f(x)=a_{0}+a_{1}x+a_{2}x^{2}+\cdots +a_{n}x^{n}$$

The coefficient $a_{0}$, $a_{1}$, $a_{2}$, …, $a_{n}$ were determined by the least squares method. The SSE and R-square values of the five-time fitting result are shown in Table 5.

**Figure 13:**
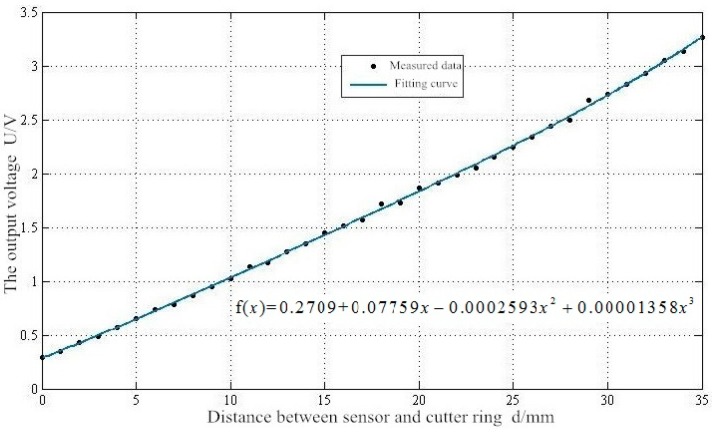
n = 3 Fitting curve.

Comparing the fitting results of five times, when n > 3, the coefficient of determination no longer increased. Therefore, the polynomial fitting of n = 3 was chosen. The fitting polynomial and curve is shown in Figure 13.

By observing the fitting curve and function in Figure 13, the following conclusions can be drawn. Considering the experimental measurement error, it can be basically determined that the linear range of the eddy current sensor designed in this study is 35 mm.

## 6. Conclusions

When the inner radius of the probe coil of the eddy current sensor is constant, the sensitivity of the eddy current sensor can be improved by increasing the number of turns or the outer radius and decreasing the thickness. The linearity of the eddy current sensor can be improved by increasing the inner radius of the probe coil. Combined with the designed circuit, the sensor probe coil parameters for measuring cutter wear were designed as inner radius 20 mm, outer radius 22 mm, wire diameter 0.2 mm, excitation frequency 1 MHz, input voltage 24 V, and output voltage 0–3.3 V.

With the developed eddy current sensor as the measuring tool, a 17-inch cutter was measured. Under the condition that edge width of the measured cutter ring was 19 mm, the designed sensor had a measuring range of 43 mm and a linear range of 33 mm. The least square method was used to fit the measured data of a 17-inch cutter, and the functional relationship between displacement and voltage was determined.

The eddy current sensor and measurement data designed in this study can meet the requirements of cutter wear measurement and be applied to the design of cutter wear monitoring system, which has certain engineering application value.

## Figures and Tables

**Table 1:** Non-contact displacement sensor characteristics.

Sensor Type	Advantages	Disadvantages	Application Situation
Laser sensor	Large range, high precision, high resolution, no requirement for the tested material	Sensitive to environmental pollutants, complex structure and high price	Nanometer displacement or vibration measurement
capacitive sensor	High resolution, small size, simple structure and low cost	Sensitivity to environmental pollutants and requirement for tested materials	Pressure sensor and acceleration sensor
eddy current sensor	Strong anti-interference ability, fast response, not affected by oil, dust, steam, etc..	Requirements for material and area to be measured	Displacement and Vibration of Large Machinery Equipment in Harsh Environment

**Table 2:** Material parameter.

Name	Cutter Ring	Sensor Coil	Solution Domain
material	40CrNiMo	copper	vacuum
Permeability $\mu_{\gamma }$ (H/m)	5000	0.999991	0
Conductivity ρ (s/m)	1,000,000	58,000,000	1

**Table 3:** Specific parameters of eddy current sensor.

Inner Radius	External Radius	Thickness	Turn Number	Line Diameter	Wall Thickness	Excitation Frequency	Rated Input Voltage	Output Voltage
20 mm	22 mm	2 mm	100	0.2 mm	3 mm	1 MHz	24 V	0～3.3 V

**Table 4:** Voltage correspondent distance.

d2/mm	U/V	d2/mm	U/V	d2/mm	U/V	d2/mm	U/V	d2/mm	U/V
0	0.29	10	0.95	20	1.87	30	2.68	40	3.29
1	0.35	11	1.02	21	1.91	31	2.74	41	3.29
2	0.43	12	1.13	22	1.99	32	2.83	42	3.29
3	0.49	13	1.17	23	2.05	33	2.93	43	3.30
4	0.57	14	1.27	24	2.15	34	3.05	44	3.30
5	0.65	15	1.35	25	2.25	35	3.13	45	3.30
6	0.74	16	1.45	26	2.34	36	3.2	46	3.30
7	0.78	17	1.51	27	2.44	37	3.26	47	3.30
8	0.87	18	1.57	28	2.50	38	3.27	48	3.30
9	0.95	19	1.72	29	2.68	39	3.28	49	3.30

**Table 5:** The fitting results.

Number of Fits	1	2	3	4	5
SSE	0.08884	0.01989	0.01478	0.01349	0.01277
R-Square	0.9967	0.9993	0.9995	0.9995	0.9995

## References

[1] Jiang Y.S. (2014). Underground Engineering Construction.

[2] Tan Q., Xia L.J., Xia Y.M. (2015). Analysis of wear rate of TBM disc cutter. J. Cent. South Univ..

[3] Dahl F., Grøv E., Breivik T. (2007). Development of a new direct test method for estimating cutter life, based on the Sievers’ J miniature drill test. Tunn. Undergr. Space Technol..

[4] Kahraman S., Fener M., Käsling H. (2016). The influences of textural parameters of grains on the LCPC abrasivity of coarse-grained igneous rocks. Tunn. Undergr. Space Technol..

[5] Wan Z.C., Sha M.Y., Zhou Y.L. (2002). Application and research of disc hob (1)—Application of TB880E TBM in Qinling tunnel construction. Mod. Tunn. Technol..

[6] Köppl F., Thuro K., Thewes M. (2015). Suggestion of an empirical prognosis model for cutting tool wear of Hydroshield TBM. Tunn. Undergr. Space Technol..

[7] Zhu R.Y., Li H.B., Zhang H.P. (2016). Research on acoustic emission signal of TBM tool based on improved CRITIC method. Shock Vib..

[8] Zhang B., Jiu J.Q., Guo W. (2013). TBM cutter wear detection device. CN. Patent.

[9] Liu Q.S., Zhang J.M., Zhang X.P. (2016). A TBM cutter wear online real-time monitoring device and method. CN. Patent.

[10] Tan Q., Zhu Z.H., Xia Y.M. (2013). A system and method for monitoring the operation status of cutter disc group of cutter-head. CN. Patent.

[11] Yang S., Hirata K., Ota T., Mitsutake Y., Kawase Y. (2015). Impedance characteristics analysis of the non-contact magnetic type position sensor. Electron. Commun. Jpn..

[12] Wang H.B. (2015). Research on Theory and Design of Sub-Nanometer Precision Eddy Current Sensor. Ph.D. Thesis.

[13] Li H.W., Liu S.Q., Yu W.T. (2011). Detection of axial displacement of maglev rotor by eddy current sensor. J. Sci. Technol..

[14] Ding T.H., Chen X.L. (2006). Eddy current sensor coil for measuring gaps between curved surfaces. J. Tsinghua Univ. (Sci. Technol.).

[15] Xia H., Cao X.R., Dong H. (2002). Eddy current sensor for measuring the speed and distance of railway locomotives. J. Harbin Eng. Univ..

[16] Wu J., Fan S.J. (2010). Design of Detection Coil Structure of Suspension Gap Sensor for Suppressing Electromagnetic Interference. J. Sens. Technol..

[17] Redko V.I., Khandetskyy V., Shembel E.M., Redko O.V., Novak P. (2011). Method and Eddy Current System for Non-Contact Determination of Interface Resistance. U.S. Patent.

[18] Jiang Z., You R., Zhang Z., Sun C. (2016). Simulation analysis on eddy current characteristics of radial core reactor. J. Magn. Mater. Devices.

[19] Zhao B., Zhang H. (2010). Application of Ansoft 12 in Engineering Electromagnetic Field.

[20] Griesbach T., Wurz M.C., Rissing L. (2013). Development, fabrication, and test of a modular eddy current micro sensor on a flexible polymer foil. Prod. Eng..

[21] Kikuchihara H., Marinova I., Saito Y. (2014). Development of a New High Sensitive Eddy Current Sensor. Mater. Sci. Forum.

[22] Yang L.J., Liu J.X., Gao S.W., Wang J.F., Xie L. (2009). Design for Large Range Electric Eddy Current Sensor. J. Instrum. Tech. Sens..

